# Predictors of Stent Restenosis in Han and Uygur Patients with Coronary Heart Disease after PCI in the Xinjiang Region

**DOI:** 10.1155/2022/7845108

**Published:** 2022-07-31

**Authors:** Jiao Wang, Yuchun Yang, Lei Zhang, Pengyi He, Huyati Mu

**Affiliations:** Department of Cardiology, First Affiliated Hospital of Xinjiang Medical University, Urumqi, China

## Abstract

**Background:**

Stent restenosis after PCI seriously affects the efficacy and prognosis; therefore, the study of ISR risk factors has become an urgent topic to be solved.

**Objective:**

To investigate the risk factors for in-stent restenosis (ISR) after percutaneous coronary intervention (PCI) in Han and Uygur patients with coronary heart disease.

**Methods:**

The clinical data of 345 Han and 127 Uygur patients who underwent intracoronary stent implantation were divided into an ISR group and a non-ISR group. The general clinical data, laboratory indicators, and coronary artery lesions were compared.

**Results:**

Age (OR = 1.040, 95% CI: 1.006∼1.075), triglycerides (OR = 1.440, 95% CI: 1.050∼1.973), total cholesterol (OR = 5.256, 95% CI: 2.826∼9.773), and ApoB (OR = 137.540, 95% CI: 11.364∼899.455) were independent risk factors for ISR after PCI in the Han patients, while ApoAI (OR = 0.002, 95% CI: 0.001∼0.011), MCV (OR = 0.824, 95% CI: 0.744∼0.911), MCH (OR = 0.421, 95% CI: 0.324∼0.548), and MCHC (OR = 0.934, 95% CI: 0.903∼0.965) were protective factors of ISR after PCI in Han patients, and the logistic regression equation composed of various factors predicted that the area under the ROC curve of ISR was 0.905. ApoB (OR = 11.571, 95% CI: 1.667∼80.340), Gensini score (OR = 1.017, 95% CI: 1.003∼1.031), and diabetes history (OR = 3.474, 95% CI: 1.189∼10.151) were independent risk factors for ISR after PCI in Uygur patients, and the area under ROC curve of ISR predicted by logistic regression equation is 0.807. The predictive efficiency of the Gensini score and ApoB level for ISR in Uygur patients was higher than that in Han, while the predictive efficiency of levels of ApoAI and MCH for ISR in Han patients was higher than that in Uygur (*P* < 0.05).

**Conclusion:**

The independent risk factors for ISR after PCI in Han and Uygur patients in Xinjiang are different.

## 1. Introduction

In recent years, the prevalence and mortality of coronary heart disease in China have been on the rise, and the control of related risk factors is limited [1, 2]. Percutaneous coronary intervention (PCI) is an important treatment for coronary heart disease [3]; however, intrastent restenosis (ISR) after PCI seriously affects the efficacy of PCI and prognosis. The incidence of ISR after metal bare stent (BMS) implantation is 15%–30%, and the widespread use of drug-eluting stents (DES) has reduced the incidence of ISR to 5%–10% [4], but this problem has not been completely solved. Therefore, it has become an urgent task to explore the risk factors for ISR and prevent them.

Xinjiang is a multiethnic region with a high incidence of coronary heart disease. Existing studies have shown that [5–7] the prevalence and mortality of coronary heart disease in those of Uygur descent in Xinjiang are higher than those of the local Han population; in addition, the incidence of three-vessel disease is higher among Uygur patients. Furthermore, the risk factors for ISR may vary among different ethnic groups.

This study was a case-control study. We retrospectively analysed the general clinical data, laboratory test results, and coronary artery lesions of 345 Han and 127 Uygur patients with coronary heart disease after PCI. The patients were divided into two groups according to the occurrence of ISR. The aim of this study was to explore the clinical characteristics and risk factors for ISR in Han and Uygur patients and to explore the predictive value of these risk factors for ISR to provide a theoretical basis for the clinical prevention of ISR.

## 2. Materials and Methods

### 2.1. Patients

From January 2019 to February 2020, a total of 3425 patients were hospitalized in the Heart Center of the First Affiliated Hospital of Xinjiang Medical University who underwent PCI and successfully implanted DES. Among them, 472 patients who came to the hospital for reexamination of coronary angiography 8–18 months after surgery were selected as the research subjects. Among the study subjects, 122 had ISR (92 Han patients and 30 Uygur patients) and 350 did not have ISR (253 Han patients and 97 Uygur patients). The ISR rate was 26.67% for the Han patients and 23.62% for the Uygur patients.

### 2.2. Inclusion and Exclusion Criteria

The inclusion criteria were as follows: (1) successfully implanted DES without poor stent adherence and (2) ISR defined as newly appearing plaques in the stent or within 5 mm from the edge of the stent after PCI, and a degree of stenosis of ≥50% of vascular proliferative disease [8]. The exclusion criteria were as follows: (1) acute myocardial infarction caused by a nonoriginal target vessel that occurred within 3 months after the first PCI and (2) patients who underwent coronary artery bypass graft surgery in the past who had a second implantation of a stent due to stenosis in the stent.

### 2.3. Data Collection

General clinical data such as age, sex, BMI, history of hypertension, history of diabetes, history of myocardial infarction, smoking history, history of double antibody and statin use after surgery, levels of uric acid, serum creatinine, portal aspartate aminotransferase (AST), alanine aminotransferase (ALT), triglycerides, total cholesterol, high-density lipoprotein cholesterol (HDL-C), low-density lipoprotein cholesterol (LDL-C), apolipoprotein AI (ApoAI), apolipoprotein B (ApoB), mean red blood cell volume (MCV), mean red blood cell haemoglobin (MCH) level, mean red blood cell haemoglobin concentration (MCHC), red blood cell distribution width (RDW), glycosylated haemoglobin (HbA1c) level, and other test results were obtained from hospital electronic medical records.

According to the standards of the American College of Cardiology [9], the number of disaffected vessels was calculated according to the involved coronary arteries, which were divided into 1, 2, and 3 lesions. When the left main artery was involved, it was calculated as 2 vessel lesions. The number of stents, the average diameter of the stents, and the total length of the stents were calculated. The Gensini score was based on the following point system [10]: stenosis <25% was scored as 1 point, 25%–49% as 2 points, 50%–74% as 4 points, 75%–90% as 8 points, 91–99% as 16 points, and 100% as 32 points. Different lesions were multiplied by different coefficients, and the final Gensini score = ∑ (coronary stenosis × lesion site coefficient).

### 2.4. Statistical Method

SASJMP 10.0 and MedCalc statistical software were used for data analysis. Quantitative data were tested by the Shapiro–Wilk test for normal distribution, conformity with normal distribution is represented by ($\bar{x}\pm s$), and the difference in mean between two independent groups was compared using the *t*-test. Nonnormally distributed data are reported as *M* (*P25*, *P75*), and the rank sum test was used for statistical analysis. The count data are expressed in absolute numbers and constituent ratios, and the chi-square test was used for analysis. With coronary stent restenosis (1 = yes, 2 = no) as the dependent variable, a multiple logistic regression analysis was carried out. A logistic regression equation was used to comprehensively predict ISR and calculate the area under the ROC curve. The area under the ROC curve was used to compare the difference in ISR predicted by different indicators among different ethnic groups.

### 2.5. Sample Size Calculation

Using the case-control sample size formula $n=2\bar{p}\bar{q}{\left(u_{a}+u_{\beta }\right)}^{2}/{\left(p_{1}-p_{0}\right)}^{2}$, when *α* = 0.05, *β* = 0.2, the restenosis rate of the Han population with better lipid control was *P*_1_=25.1%, and the restenosis rate of the Han population with poor lipid control was *P*_0_=35.8%, *n* = 165; a total of 330 Han people were needed. The restenosis rate of Uygur with better lipid control was *P*_1_=25.7%, and the restenosis rate of Uygur with poor control was *P*_0_=40.8%, *n* = 68; a total of 136 Uygur people were needed.

## 3. Result

### 3.1. Comparison of Clinical Data between the Han and Uygur ISR and Non-ISR Groups

The incidence of hypertension and diabetes history in the Han ISR group was significantly higher than that in the non-ISR group, and the difference was statistically significant (Table 1) (*P*=0.041, *P* < 0.001). The incidence of diabetes history in the Uygur ISR group was significantly higher than that in the non-ISR group, and the difference was statistically significant (*P*=0.002). The levels of total cholesterol, LDL-C, and glycosylated haemoglobin in the Han ISR group were higher than those in the non-ISR group (*P*=0.002, *P* < 0.001, *P* < 0.001), and the levels of ApoAI and MCH were lower than those in the non-ISR group (all *P* < 0.001); all these differences were significant. The ApoB level of the Uygur ISR group was higher than that of the non-ISR group, the MCV level was lower than that of the non-ISR group, and the difference was significant (*P*=0.025, *P*=0.041).

### 3.2. Comparison of Coronary Artery Lesions in the ISR and Non-ISR Groups of the Han and Uygur Patients

The proportion of three-vessel lesions in the Han ISR group was significantly higher than that in the non-ISR group. The proportion of single-vessel and double-vessel lesions was lower than that in the non-ISR group, and the difference was significant (Table 2) (*P*=0.035). The Gensini score of the Uygur ISR group was significantly higher than that of the non-ISR group, and the difference was significant (*P*=0.004).

### 3.3. Logistic Regression Analysis of ISR Risk Factors in Han and Uygur Patients and the ROC Curve of the Regression Equation to Predict Restenosis

**Figure 1:**
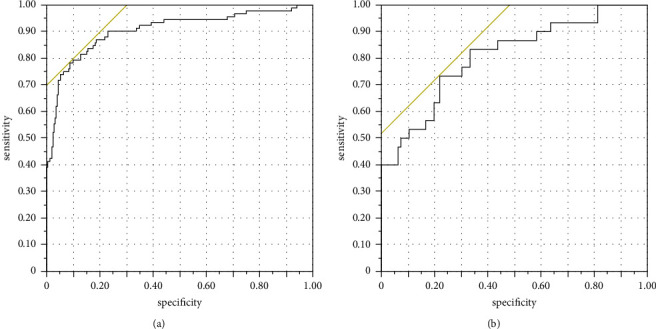
Logistic regression predicts the ROC curve of restenosis. (a) Han nationality, AUC = 0.905; (b) Uygur nationality, AUC = 0.807.

Multivariate logistic regression analysis of the Han patients showed that age (*OR* = 1.040, *95%CI*: 1.006∼1.075), triglycerides (OR = 1.440, 95% CI: 1.050∼1.973), total cholesterol (OR = 5.256, 95% CI: 2.826∼9.773), and ApoB (OR = 137.540, 95% CI: 11.364∼899.455) were independent risk factors for ISR in the Han patients after PCI and that ApoAI (OR = 0.002, 95% CI: 0.001∼0.011), MCV (OR = 0.824, 95% CI: 0.744∼0.911), MCH (OR = 0.421, 95% CI: 0.324∼0.548), and MCHC (OR = 0.934, 95% CI: 0.903∼0.965) were protective factors for ISR in the Han patients after PCI (Table 3 and Figure 1). The logistic regression equation was composed of the above factors [*y* = −12.987 + 0.039 age + 0.364 triglycerides + 1.659 total cholesterol−6.473 ApoAI + 4.924 ApoB − 0.194 MCV − 0.865 MCH − 0.068 MCHC], and the area under the ROC curve for predicting ISR was 0.905 (Figure 1).

Multivariate logistic regression analysis of the Uygur patients showed that ApoB (OR = 11.571, 95% CI: 1.667∼80.340), Gensini score (OR = 1.017, 95% CI: 1.003∼1.031), and history of diabetes (OR = 3.474, 95% CI: 1.189∼10.151) were independent risk factors for ISR in these patients after PCI (Table 3). The logistic regression equation was composed of the above factors [*y* = −11.202 + 2.449 ApoB + 0.017 Gensini score + 0.623 diabetes history], and the area under the ROC curve for predicting ISR was 0.807 (Figure 1).

### 3.4. The Difference ROC Curve of Each Index Predicting ISR in the Han and Uygur Patients

**Figure 2:**
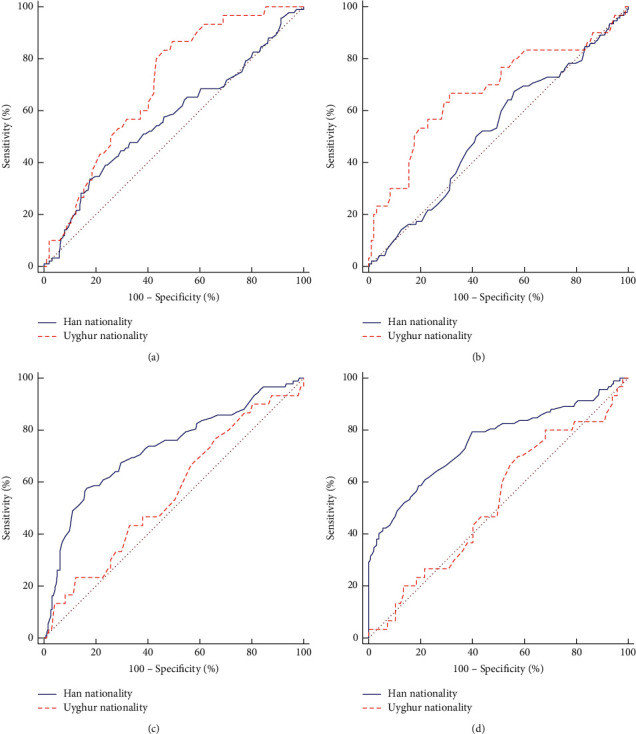
Different ROC curves for predicting ISR by different indexes in Han and Uygur patients. (a) Gensini score; (b) ApoB; (c) MCH; (d) ApoAI.

The Gensini score, ApoB, ApoAI, and MCH had different predictive efficacies for ISR in the Han and Uygur patients, and their cutoff value, sensitivity, and specificity were significantly different (Table 4 and Figure 2). The predictive efficacy of the Gensini score (AUC = 0.696, cutoff value 56, sensitivity 86.67%, specificity 50.52%) and ApoB (AUC = 0.676, cutoff value 0.92, sensitivity 66.67%, specificity 68.75%) in the Uygur patients was higher than that in the Han patients, and the predictive efficacy of ApoAI (AUC = 0.754, cutoff value 1.02, sensitivity 79.35%, specificity 60.08%) and MCH (AUC = 0.729, cutoff value 29.3, sensitivity 57.61%, specificity 83.34%) in the Han patients was higher than that in the Uygur patients, and the difference in the AUC was significant (all *P* < 0.05).

## 4. Discussion

The formation of ISR is influenced by many factors, and its pathophysiological mechanisms include vascular endothelial injury, smooth muscle cell proliferation, inflammation, arterial wall remodelling, and so on [11]. Previous studies have shown that [12] poor control of blood lipids, especially substandard LDL-C concentration, is an independent risk factor for ISR after PCI treatment. At present, the optimal LDL-C level after PCI has been controversial, even the concept of target, threshold, or target LDL-C level [13]. ApoB and ApoAI are used as apolipoproteins of LDL-C and HDL-C, respectively, which can directly affect lipid metabolism. The content of ApoB in the blood can reflect the number of atherogenic particles in the body [14]. ApoAI is an antiatherosclerotic factor involved in the reverse transport of cholesterol [15]. This study found that the total cholesterol and LDL-C levels of the Han ISR group were higher than those of the non-ISR group and that the ApoAI level was lower than that of the non-ISR group. The ApoB level of the Uygur ISR group was higher than that of the non-ISR group. Further multivariate regression analysis found that triglycerides, total cholesterol, and ApoB were independent risk factors for ISR in the Han population in Xinjiang, that ApoAI was a protective factor for ISR in the Han population, and that ApoB was also an independent risk factor for ISR in the Uygur patients in Xinjiang. This is similar to previous research results suggesting that even in patients of different ethnicities, poor blood lipid control, especially high levels of ApoB, may be a risk factor for ISR but that high ApoAI levels may be a protective factor for ISR, which provides a clinical basis for blood lipid management in patients after PCI. This study also demonstrated that the risk factors for ISR in blood lipids were not identical between the two ethnic groups, which may be related to the long-term differences in living and eating habits between different ethnic groups as well as the individualized differences in postoperative statin therapy.

A study by Nanjing [16] found that there were differences in MCV, MCH, and MCHC levels between the ISR group and the non-ISR group, but the predictive value of these three indicators for ISR was limited. In this study, it was found that the MCH level in the Han ISR group was lower than that in the non-ISR group and that the MCV level in the Uygur ISR group was lower than that in the non-ISR group. Further multivariate regression analysis showed that MCV, MCH, and MCHC were protective factors for ISR in Han patients. These three red blood cell parameters have been found to be associated with a variety of diseases in recent years, including cardiovascular disease. Considering that the low levels of these three indicators may indicate an iron deficiency state in the body, this state leads to an increase in the inflammatory response in the body [17], which may influence the development of ISR. The results for the Han population in this study are not identical to those of the previous study, which may be related to the regional differences of the included population. Moreover, both studies are single-centre studies and have the problem of limited sample size, so further expansion of the sample size or multicentre studies is needed for verification.

This study found that the Gensini score and a history of diabetes were independent risk factors for ISR in the Uygur patients after PCI. Univariate analysis indicated that the diabetes history in the ISR groups of the two nationalities was significantly higher than that of the non-ISR group of the same nationality and that the HbA1c level in the Han ISR group was higher than that in the non-ISR group. This is consistent with previous foreign guidelines [18]: diffuse vascular lesions in patients with cardiovascular disease complicated by diabetes greatly increase the possibility of ISR after PCI, and the risk of venous bridge occlusion after coronary artery bypass graft is also higher.

Although previous studies [19] have shown that type of stent implantation, diabetes, prior bypass surgery, and small vessel diameter are predictors of ISR. However, this study found that the independent risk factors for ISR in different ethnic groups were not the same and that there were also some protective factors. At the same time, previous studies have shown that [20] the ratio of monocytes to high-density lipoprotein (MHR) as a new kind of inflammatory marker, combined with the interaction between two kinds of risk factors and protective factors, has a certain predictive value for ISR in patients with premature coronary heart disease (PCHD), and its prediction value is higher than that of mononuclear cells or high-density lipoprotein cholesterol alone predictive value. In conclusion, considering that a single risk factor cannot fully explain the occurrence of ISR, a logistic regression equation was established to observe its predictive value for ISR after PCI. The results indicated that the area under the ROC curve predicted by the logistic regression equation for the Han patients was 0.905 and that for the Uygur group was 0.807. These results indicate that the logistic regression equation composed of various factors may serve as a potential predictor of ISR after PCI in Han and Uygur patients in Xinjiang. At the same time, this study also found that there were ethnic differences in the predictive efficacy of different indicators for ISR in the Han and Uygur patients.

In summary, many factors, such as poor blood lipid control, red blood cell parameters, combined diabetes history, high Gensini score, and other factors, participate in the formation of ISR. The logistic regression equation composed of the respective risk factors and protective factors of different populations can better predict the occurrence of ISR after PCI.

## Figures and Tables

**Table 1:** Comparison of clinical data between Han and Uygur ISR and non-ISR patients ($\bar{x}\pm s$, *n* (%), *M* (*P*25, *P*75).

Characteristic	Han ISR group	Han non-ISR group	*χ* ^2^ */t*	*P*	Uygur ISR group	Uygur non-ISR group	*χ* ^2^ */t*	*P*
	*n* = 92	*n* = 253			*n* = 30	*n* = 97		
Sex (male *n* (%)/female *n* (%))	66 (71.74)/26 (28.26)	188 (74.31)/65 (25.69)	0.229	0.632	26 (86.67)/4 (13.33)	75 (77.32)/22 (22.68)	1.23	0.268
Age (years)	63.1 ± 11.24	60.45 ± 10.63	1.966	0.051	61.03 ± 7.55	58.65 ± 9.73	1.406	0.165
BMI (kg/m^2^)	25.48 ± 3.37	25.76 ± 2.79	0.729	0.467	29.66 ± 3.33	28.34 ± 3.76	1.843	0.071
History of hypertension (*n* (%))	68 (73.91)	157 (62.06)	4.182	0.041	21 (70)	61 (62.89)	0.507	0.477
History of diabetes (*n* (%))	56 (60.87)	78 (30.83)	25.629	＜0.001	17 (56.67)	25 (25.77)	9.88	0.002
History of myocardial infarction (*n* (%))	19 (20.65)	61 (24.11)	0.453	0.501	4 (13.33)	10 (10.31)	0.214	0.644
Smoking history (*n* (%))	42 (45.65)	119 (47.04)	0.052	0.82	16 (53.33)	49 (50.52)	0.073	0.787
Take double antibodies regularly (*n* (%))	70 (76.09)	199 (78.66)	0.259	0.611	26 (86.67)	83 (85.57)	0.023	0.88
Take statins regularly [*n* (%)]	76 (82.61)	216 (85.38)	0.397	0.529	26 (86.67)	85 (87.63)	0.019	0.89
Uric acid	349.98 (297.75, 391.2)	339.01 (277.71, 394.86)	1.558	0.212	340.01 (290.68, 409.99)	342 (297.53, 400.23)	0.003	0.961
Serum creatinine	75.89 (63.66, 86.34)	74.14 (62.71, 85.5)	0.716	0.398	78.79 (66.02, 91.64)	76.61 (66.7, 86.88)	0.174	0.677
AST	24.47 (19.55, 31.6)	24.2 (18.9, 29.4)	0.001	0.974	21.57 (16.3, 26.31)	22.3 (17.54, 26.35)	0.613	0.434
ALT	23.91 (17.47, 34.03)	25.58 (16.8, 33.42)	0.884	0.347	20.25 (15.93, 32.72)	25.2 (18.56, 33.43)	0.613	0.434
Triglycerides	1.42 (0.98, 2.14)	1.38 (0.97, 2.02)	0.06	0.807	2.16 (1.2, 2.78)	1.5 (1.04, 2.17)	2.937	0.087
Total cholesterol	3.49 (2.91, 3.91)	3.02 (2.52, 3.48)	9.49	0.002	3.59 (2.99, 4.3)	3.11 (2.67, 3.79)	2.937	0.087
HDL-C	0.92 (0.78, 1.1)	0.96 (0.8, 1.1)	0.017	0.896	0.85 (0.75, 1.05)	0.86 (0.74, 1.07)	0.138	0.71
LDL-C	2.28 (1.76, 2.76)	1.71 (1.35, 2.16)	26.403	＜0.001	1.96 (1.52, 2.91)	1.84 (1.45, 2.34)	0.217	0.642
ApoAI	0.88 (0.67, 1.02)	1.08 (0.96, 1.21)	41.702	＜0.001	0.98 (0.93, 1.14)	0.98 (0.9, 1.11)	0.008	0.928
ApoB	0.78 (0.7, 0.85)	0.71 (0.59, 0.85)	0.443	0.506	1.03 (0.76, 1.24)	0.79 (0.62, 0.97)	5.045	0.025
MCV	92.35 (89.4, 95.6)	92.5 (89.35, 94.9)	0.15	0.698	89.2 (87.75, 91.58)	90.8 (88.1, 93.35)	4.194	0.041
MCH	29 (28.1, 30.7)	30.8 (29.7, 32)	28.767	＜0.001	29.95 (28.7, 30.43)	30 (28.85, 31.1)	0.071	0.79
MCHC	336 (330.25, 344.75)	337 (329, 345)	1.007	0.316	334 (328.75, 343.25)	335 (327, 343)	0.627	0.429
RDW	13 (12.43, 13.4)	12.8 (12.3, 13.3)	3.09	0.079	13.2 (12.7, 13.7)	13.2 (12.55, 13.75)	0.123	0.726
HbA1c	7.25 (6.3, 8.1)	6.2 (5.8, 6.9)	26.259	＜0.001	6.63 (6, 7.93)	6.3 (5.85, 6.9)	1.78	0.182

**Table 2:** Comparison of coronary artery lesions between Han and Uygur patients with ISR and non-ISR (*M* (*P*25, *P*75), *n* (%).

Coronary artery lesions		Han ISR group	Han non-ISR group	*χ* ^*2*^	*P*	Uygur ISR group	Uygur non-ISR group	*χ* ^*2*^	*P*
		*n* = 92	*n* = 253			*n* = 30	*n* = 97		
Number of coronary artery lesions (*n* (%))	1	10 (10.87)	53 (20.95)	6.732	0.035	2 (6.67)	17 (17.53)	2.154	0.341
	2	25 (27.17)	79 (31.23)			10 (33.33)	30 (30.93)		
	3	57 (61.96)	121 (47.82)			18 (60.00)	50 (51.54)		

Number of stents (*n* (%))	1	37 (40.21)	125 (49.40)	3.791	0.285	11 (36.67)	50 (51.55)	2.133	0.545
	2	31 (33.70)	62 (24.51)			7 (23.33)	17 (17.53)		
	3	13 (14.13)	41 (16.21)			7 (23.33)	16 (16.49)		
	4 or more	11 (11.96)	25 (9.88)			5 (16.67)	14 (14.43)		

Stent diameter *M* (*P*_*25*_, *P*_*75*_)		2.75 (2.5, 3)	2.75 (2.5, 3.01)	2.18	0.14	2.78 (2.61, 3)	2.7 (2.5, 2.81)	1.684	0.194

Stent length *M* (*P*_*25*_, *P*_*75*_)		44.5 (30, 69)	38 (28, 69)	2.575	0.109	59.5 (30, 84.5)	38 (24, 77.5)	2.062	0.151

Gensini score *M* (*P*_*25*_, *P*_*75*_)		66 (39.25, 92)	54 (40, 78)	3.146	0.076	78.5 (62, 94.5)	55 (27.5, 80)	8.368	0.004

**Table 3:** Logistic regression analysis of risk factors for ISR after PCI in Han and Uygur patients.

Risk factor	*B*	*SE*	*Wald*	*P*	*OR*	*95% CI*
Han nationality						
Constant	−12.987	6.129	4.49	0.034		
Age	0.039	0.017	5.567	0.018	1.04	1.006–1.075
Triglycerides	0.364	0.161	6.152	0.013	1.44	1.050–1.973
Total cholesterol	1.659	0.316	32.354	＜0.001	5.256	2.826–9.773
ApoAI	−6.473	0.999	69.845	＜0.001	0.002	0.001–0.011
ApoB	4.924	1.274	18.534	＜0.001	137.54	11.360–899.455
MCV	−0.194	0.052	19.006	＜0.001	0.824	0.744–0.911
MCH	−0.865	0.134	61.965	＜0.001	0.421	0.324–0.548
MCHC	−0.068	0.017	18.9	＜0.001	0.934	0.903–0.965

Uygur nationality						
Constant	−11.202	4.27	6.88	0.009		
ApoB	2.449	0.989	6.471	0.011	11.571	1.667–80.340
Gensini score	0.017	0.007	5.578	0.018	1.017	1.003–1.031
History of diabetes	0.623	0.274	2.253	0.022	3.474	1.189–10.151

**Table 4:** The difference in the area under the curve for ISR predicted by different indicators in Han and Uygur.

Risk factor	Nationality	Cutoff value	Sensitivity (%)	Specificity (%)	AUC	*95%CI*	*Z*	*P*
Gensini score	Han	86	33.70	82.21	0.566	0.512–0.619	2.112	0.034
	Uygur	56	86.67	50.52	0.696	0.608–0.775

ApoB	Han	0.74	67.39	43.87	0.522	0.468–0.576	2.142	0.032
	Uygur	0.92	66.67	68.75	0.676	0.587–0.757

ApoAI	Han	1.02	79.35	60.08	0.754	0.705–0.798	3.336	<0.001
	Uygur	0.96	70.00	42.27	0.523	0.433–0.613

MCH	Han	29.3	57.61	83.34	0.729	0.678–0.775	2.491	0.013
	Uygur	30.4	76.67	34.02	0.557	0.466–0.645

## Data Availability

The datasets used and/or analysed during the current study are available from the corresponding author on reasonable request.

## References

[1] Diseases N. C. F. C. (2019). *Chinese Cardiovascular Health and Disease Report*.

[2] Jiang G., Wang D., Li W. (2012). Coronary heart disease mortality in China: age, gender, and urban-rural gaps during epidemiological transition. *Revista Panamericana de Salud Públic*.

[3] Moulias A., Alexopoulos D. (2011). Long-term outcome of percutaneous coronary intervention: the significance of native coronary artery disease progression. *Clinical Cardiology*.

[4] Kim M. S., Dean L. S. (2011). In-stent restenosis. *Cardiovascular therapeutics*.

[5] Huang W. J., Liu J. M., Xie W. (2012). Clinical study of coronary artery disease in Han, Uygur and Kazak patients. *Chinese Journal of Interventional Cardiology*.

[6] Ma Y. T., Liu Y., Tang B. P. (2001). Comparison of coronary angiography between Uygur and han in Xinjiang. *Chinese Journal of Interventional Cardiology*.

[7] Jia W. X., Zhang Q. N., Sun X. R. (1994). A comparative study of coronary artery angiography between Uygur and Han in Xinjiang. *Chinese Journal of Radiology*.

[8] Hee L., Terluk A., Thomas L. (2017). Late clinical outcomes for sequent please paclitaxel-coated balloons in PCI of instent restenosis and de novo lesions: a single-center, real world registry. *Catheterization and Cardiovascular Interventions*.

[9] Austen W. G., Edwards J. E., Frye R. (1975). A reporting system on patients evaluated for coronary artery disease. Report of the ad hoc committee for grading of coronary artery disease, council on cardiovascular surgery, American heart association. *Circulation*.

[10] Gensini G. G. (1983). A more meaningful scoring system for determining the severity of coronary heart disease. *American Journal of Cardiology*.

[11] Looser P. M., Kim L. K., Feldman D. N. (2016). In-stent restenosis: pathophysiology and treatment. *Current Treatment Options in Cardiovascular Medicine*.

[12] Zhao C. R., Bai M., Zhang B. (2015). The relationship between blood lipid control level and stent restenosis after coronary stent implantation. *Chinese Journal of Circulation*.

[13] Martin G. J., Teklu M., Mandieka E., Feinglass J. (2022). Low-density lipoprotein cholesterol levels in coronary artery disease patients: opportunities for improvement. *Cardiology Research and Practice*.

[14] Welsh C., Celis-Morales C. A., Brown R. (2019). Comparison of conventional lipoprotein tests and apolipoproteins in the prediction of cardiovascular disease. *Circulation*.

[15] Yang P. B., Huang G. Y., Wang Y. (2019). Clinical significance of serum ApoB/ApoA1 ratio in the diagnosis of atherosclerosis. *Journal of PLA Medical College*.

[16] Chen C., Jin Z. N., Peng J. J. (2020). Relationship between erythrocyte parameters and stent restenosis after coronary stent implantation. *Chinese Journal of General Practice*.

[17] Shah A. S. (2017). Iron: its complicated. *Journal of Heart and Lung Transplantation*.

[18] Rydén L., Grant P. J., Anker S. D. (2013). ESC guidelines on diabetes, pre-diabetes, and cardiovascular diseases developed in collaboration with the EASD: the task force on diabetes, pre-diabetes, and cardiovascular diseases of the European society of cardiology (ESC) and developed in collaboration with the European association for the study of diabetes (EASD). *European Heart Journal*.

[19] Ullrich H., Olschewski M., Münzel T., Gori T. (2021). Coronary in-stent restenosis: predictors and treatment. *Deutsches Ärzteblatt International*.

[20] Chen Bo-W., Liu J.-J., Xing J.-H. (2022). Analysis of the correlation between the ratioof monocytes to high-density lipoproteincholesterol and in-stent restenosis in patientswith premature coronary heart disease. *Clinical and Applied Thrombosis*.

